# Supplementary material for: Consistent Biomarkers and Related Pathogenesis Underlying Asthma Revealed by Systems Biology Approach
Source: Int J Mol Sci. 2019 Aug 19;20(16):4037. doi: 10.3390/ijms20164037 (PMC6720652; doi:10.3390/ijms20164037)
Supplement: Supplementary file 1 [file ijms-20-04037-s001.zip › ijms-566768-supplementary-XML/Supplementary Material S2-Reports of Quality control for ten datasets.zip!/Supplementary Material S2-Reports of Quality control for ten datasets/QC_report_for_GSE18965/hm.pdf]

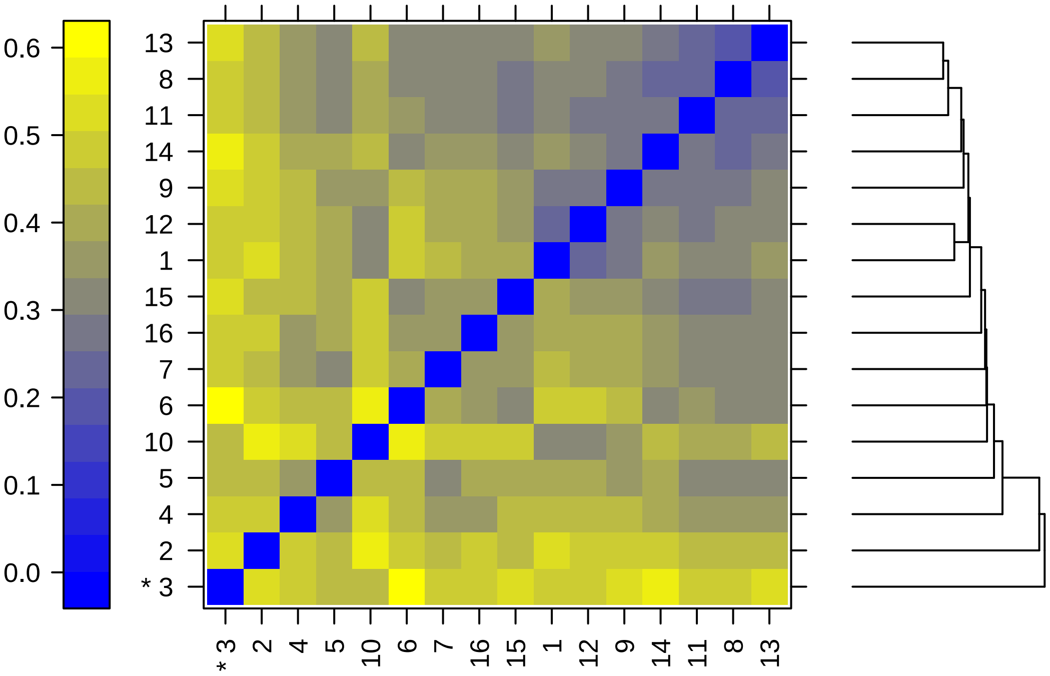

0.6
0.5
0.4
0.3
0.2
0.1
0.0
13
8
11
14
9
12
1
15
16
7
6
10
5
4
2
* 3
* 3
2
4
5
10
6
7
16
15
1
12
9
14
11
8
13
